# Key factors in work engagement and job motivation of teaching faculty at a university medical centre

**DOI:** 10.1007/s40037-013-0080-1

**Published:** 2013-09-14

**Authors:** B. A. M. van den Berg, Arnold B. Bakker, Th. J. ten Cate

**Affiliations:** 1 Department of Educational Development and Training, Faculty of Social and Behavioural Sciences, Utrecht University, Heidelberglaan 1, 3584 CS Utrecht, the Netherlands; 2 Department of Work and Organizational Psychology, Erasmus University Rotterdam, Rotterdam, the Netherlands; 3 Center for Research and Development of Education, University Medical Center Utrecht, Utrecht, the Netherlands

**Keywords:** Teaching motivation, Teaching workload, Roles of teacher, Management and/or staff development, Staff workload

## Abstract

This study reports about teacher motivation and work engagement in a Dutch University Medical Centre (UMC). We examined factors affecting the motivation for teaching in a UMC, the engagement of UMC Utrecht teaching faculty in their work, and their engagement in teaching compared with engagement in patient care and research. Based on a pilot study within various departments at the UMCU, a survey on teaching motivation and work engagement was developed and sent to over 600 UMCU teachers. About 50 % responded. Work engagement was measured by the Utrecht Work Engagement Scale, included in this survey. From a list of 22 pre-defined items, 5 were marked as most motivating: teaching about my own speciality, noticeable appreciation for teaching by my direct superior, teaching small groups, feedback on my teaching performance, and freedom to determine what I teach. Feedback on my teaching performance showed the strongest predictive value for teaching engagement. Engagement scores were relatively favourable, but engagement with patient care was higher than with research and teaching. Task combinations appear to decrease teaching engagement. Our results match with self-determination theory and the job demands–resources model, and challenge the policy to combine teaching with research and patient care.

## Introduction

Teacher motivation is important both because it affects the investment of energy in teaching and presumably its quality, but also because motivated faculty are likely to engage students in investing energy in studying, independent of the quality of their teaching [1].

Teaching in academic medical settings often implies the combination of various obligations. Physicians have patient care responsibilities, and both clinical and basic science faculty have research tasks. As health care is increasingly regulated with time-on-task requirements and university centres are dependent on research productivity and funding, teaching tasks may find themselves squeezed between competing obligations which might contribute to a decrease in satisfaction [2]. Research and patient care both benefit from more tangible outcomes than teaching. This is one reason why career advancement based on teaching is less clear cut than when it is based on research or patient care. In addition, medical curricula have become more integrated, are frequently executed in small-group settings and increasingly rely on teaching skills other than didactic lecturing to convey knowledge. Given the limits of time to engage in teaching, high quality education must draw on considerable motivation of medical faculty.

DaRosa et al. [3] recently delineated the many barriers to effective teaching that faculty in medical schools face. These may range from curriculum barriers (unclear objectives, curriculum structure) to cultural barriers (student and faculty attitudes) and environmental and financial barriers. As an example of cultural barriers in students, DaRosa and colleagues mention students preferring lectures over instructional strategies that require active learning. A cultural barrier in faculty may be an attitude of seeing teaching as a distraction from patient care. Environmental and financial barriers include a lack of time and resources and too little access to patients and teaching support. One element that could be added to DaRosa et al.’s recommendations to restore the conditions for high quality teaching, is to support teacher motivation.

Motivation for teaching can be understood using self-determination theory (SDT). According to SDT [4, 5], apart from physical needs, human behaviour is guided by three basic psychological needs: a need for self-determination (designated as ‘Autonomy’), a need to feel capable (called ‘Competence’), and the need for relatedness (‘Relatedness’). Research in the field of work and organizational psychology builds on these insights. Since most people work for a substantial part of their lives, these basic needs should be fulfilled for a large part through work. Satisfaction of needs, as defined within SDT, can explain interrelations between job demands, job resources and work engagement. Van den Broeck et al. [6] suggest that employees experiencing resourceful job characteristics that address these needs feel more motivated and engaged in their work.

Using the job demands–resources model [7], job resources (such as job control, opportunities for learning, and support from co-workers and superiors) have been found to be positively associated with work engagement, leading to better performance [8]. These resources not only have a buffering effect on the energy depleting effects of work, but also stimulate work motivation. By ensuring sufficient and appropriate job resources for all employees, the organization can make an important contribution to job satisfaction and engagement. Next to general psychological needs that job resources can fulfil, each type of work has its own specific job stressors, which ask for appropriate energy sources to buffer them. A recent qualitative investigation of career satisfaction in medical faculty in Brazil [9] suggested that teacher responsibility and autonomy in decision-making, the learning of new skills in faculty development, expectation of professional growth, recognition, and reinforcement are some of the key factors.

Our study looks at discerning job resources that enhance the motivation of medical teachers. We investigated the engagement of medical teachers working in the University Medical Center Utrecht (UMCU) in their teaching task, in comparison with their engagement in competing tasks of research and patient care, to determine routes to increase teacher motivation in the academic medical environment.

Specific questions were: (1) Which factors affect the motivation for teaching in a UMC? (2) How engaged is UMCU teaching faculty in their work? (3) How does the engagement of UMCU teaching faculty in teaching compare with their engagement for patient care and research tasks?

## Methods

### Pilot study

To uncover relevant factors that may affect teaching motivation of faculty at UMCU, we first developed a semi-structured interview guide based on topics in the literature on Self-Determination and Work Engagement. The main themes were the importance for the interviewee of: appreciation of colleagues and management for teaching, feeling of relatedness with colleagues, autonomy to take one’s own decisions, practical support, and opportunities for a teaching career. Interviews were conducted with 16 faculty, most of them combining teaching with patient care or research tasks. Interviewers were the first author and a second educationalist from Utrecht University (see acknowledgments). Interviewees were clinical and non-clinical faculty, differing in teaching experience and seniority, from various disciplines within UMCU. All respondents were asked permission to record the interviews and were informed that personal data would not be shared with others than the researchers.

All interviews were independently analyzed by two researchers, focusing on the importance the respondents themselves attached to the themes introduced by the interview questions. New factors that appeared to affect motivation for teaching were collaboratively identified by the research team from the interview data.

### Survey

The pilot study yielded 22 items about factors that might affect teaching motivation, These items were included in an electronic survey and had to be scored on a 5-point Likert scale ranging from 1 (very negative) to 5 (very positive). To measure the work engagement of UMC teachers, the Utrecht Work Engagement Scale (UWES-9) was integrated in this survey.

The UWES-9 is a validated self-report questionnaire developed by Schaufeli et al. [10]. This instrument measures work engagement, a concept with a more active, energetic connotation than job satisfaction [4, 10]. UWES-9 was chosen because of its possibility to compare occupational groups [11]. Work engagement can be defined as ‘a positive work-related state of fulfilment that is characterized by vigour, dedication, and absorption’. Vigour is characterized by high levels of energy and mental resilience while working, the willingness to invest effort in one’s work, and persistence even in the face of difficulties. Dedication refers to being strongly involved in one’s work and experiencing a sense of significance, enthusiasm, inspiration and challenge. Absorption is characterized by being fully concentrated and happily engrossed in one’s work, whereby time passes quickly and one has difficulty to detach from work.

We used UWES-9 not only to measure work engagement for the job as a whole, but also for the three separate work activities of teaching, research and patient care. To make this possible the instrument was slightly modified, in agreement with the developers.

Respondents were also asked to indicate their UMCU-Division of employment, age, years of teaching experience, teaching programme and number of teaching hours annually. Finally, they were invited to add items they felt were missing as factors affecting teacher motivation and to add any remarks to teaching motivation they felt to be relevant.

The questionnaire was distributed via directors of UMCU educational programmes and courses. They were asked to forward an e-mail request to all teachers in their programmes to fill out the electronic questionnaire. This way, the questionnaire reached the addresses of over 600 teachers, covering most of the UMCU teaching faculty. We considered teaching faculty those clinical and basic science faculty involved in academic courses (i.e. excluding non-academic teaching) in so far as they were known by course and rotation coordinators. The survey was administered anonymously. In a small minority of cases, which could not be fully documented, a teacher received the request twice. The data collection was conducted between November 2010 and early 2011 and completed on 18 February 2011.

The quantitative data were analyzed using descriptive statistics; differences between groups of respondents were tested using parametric and nonparametric tests. The qualitative data from the open questions were first independently classified by the first author and a third researcher (see acknowledgements), after which classifications were compared and discussed. This led to categories that enabled the ordering of comments.

## Results

### Response analysis

Of all approached UMCU teachers, 376 opened the questionnaire. Respondents who stopped answering after the first two questions (70) were removed from the dataset. It is not clear why they stopped. Only two respondents indicated they did not agree with the use of their data for research. We estimate the remaining group of respondents (306) to be around 50 % of the intended population. This is not unsatisfactory, considering that the relevant persons were not directly contacted by e-mail, but through third persons. Enquiries revealed that some teachers were never reached. In some cases we learned that the request to forward questionnaires had simply ‘escaped the attention’.

The age of 42 % of the respondents was under 40, 21 % between 40 and 50 and 33 % over 50. They differed in years of teaching experience, 32 % having taught for 5 years or less, 23 % 5–10 and 40 % more than 10 years. Half of them (54 %) reported teaching 4 h or less per week, half or them (46 %) more than 4 h. Most respondents (80 %) indicated they combined teaching duties with other tasks, often with research and patient care (49 %), less frequently with research only (22 %) or with patient care only (8 %). 50 % of the responses came from divisions with a high teaching load (Table 1). Divisions include departments (e.g. neurological and psychiatric specialities reside under the neuro-sciences division; ENT is subsumed under surgical specialities); the division names have been translated freely to give the reader a sense of which departments they contain.

**Table 1:** Responses per division of employment, teaching load per division

UMCU divisions	Response (*N* = 306)	Teaching load^a^
Internal medicine and dermatology	43	+
Neuro sciences	34	+
Family medicine, public health and epidemiology	28	+
Education department	30	+
Surgical specialities	18	+
Paediatrics	39	
Lab and pharmacy	26	
Lung and cardiac specialities	14	
Gynaecology, obstetrics and neonatology	13	
Biomedical and genetic sciences	11	
Perioperative and emergency care	11	
Imaging	10	
Division not mentioned	28	

^a^UMCU divisions were grouped based on the number of paid teaching hours for the medical curriculum of 2009–2010. ‘+’ signifies a relatively high teaching load

### Motivating factors

Table 2 shows ranked mean scores and standard deviation of scores on the 22 items about factors that may affect teaching motivation. Highly ranked motivators for the teaching task were ‘Teaching about my own speciality’; ‘Noticeable appreciation for teaching by my direct superior’; ‘Teaching to small groups (~12 students)’; ‘Feedback on my teaching performance’, and ‘Freedom to determine what I teach’. The motivators that score lowest relate to the opportunity to do research on teaching, and teaching to large and medium sized groups. These factors still all show scores higher than the average of 3.0 on a 5-point scale.

**Table 2:** Mean scores on motivating factors to teaching (*N* = 306)

	M^a^	SD
Teaching about my own speciality	4.50	0.56
Noticeable appreciation for teaching from my direct superior	4.10	0.70
Teaching small groups (~12 students)	4.06	0.80
Feedback on my teaching performance	4.03	0.60
Freedom to determine what I teach	4.03	0.64
Noticeable appreciation for teaching of my immediate colleagues	3.99	0.67
Teaching in which the transfer of content is paramount	3.98	0.66
Freedom to determine how I teach	3.94	0.65
More secretarial assistance in my educational task	3.79	0.72
Teaching with colleagues from other disciplines	3.75	0.75
Numerical rating/scores from student evaluations	3.73	0.74
Give more publicity to good teachers	3.72	0.70
Financial reward for obtaining a teaching qualification	3.70	0.81
Feedback from other teachers or teacher teams	3.68	0.67
Easing procedures of basic and senior teaching qualification	3.63	0.84
Wider availability of teacher training	3.62	0.71
Teaching with emphasis of the learning process	3.57	0.96
More educational assistance from the UMCU Education department	3.54	0.69
The possibility of a teaching career	3.54	0.82
Teaching medium sized groups (about 40–60 students)	3.38	0.86
Teaching large groups (in the lecture hall)	3.30	0.94
Doing research and publishing on education	3.03	0.79

^a^Values: 1 (low) to 5 (high)

Respondents added 306 items in reaction to the open question: ‘What stimulates your motivation in teaching?’ and 292 items in reaction to the open question: ‘What hampers your motivation in teaching?’ Items interpreted to fall in these categories are listed in Table 3. ‘Motivated students’ stands out, as it is mentioned frequently as a stimulating factor when present, or as a hampering factor when it lacks. Time planning and sufficient facilities are other frequently mentioned items that motivate or demotivate when lacking.

**Table 3:** Additional factors named to stimulate or hamper teaching motivation

Factors that stimulate my motivation for teaching	*N*	Factors that hamper my motivation for teaching	*N*
Motivated students	62	Unmotivated students	52
Acknowledgment for my teaching	33	Poor facilities	52
Adequate time planning of teaching	32	Too little time provided to properly teach	50
Clear organization and expectations	32	Bureaucracy and rules around teaching	51
Adequate teaching facilities	27	Not sufficiently familiar with content	15
Application of my content knowledge	24	Monotonous repeated group teaching	8
Receiving feedback on my teaching	23		
Teaching fixed groups over time	17		
Having personal contacts with students	17		
Other items	29	Other items	74
Total	306	Total	292

### Work engagement

The work engagement of UMC teaching faculty for the combination of tasks was on average 4.26 of a 6-point frequency scale (SD 0.85). This figure differs per task: respondents indicated they felt a higher engagement with patient care (4.42) than with research (3.86), and education (3.79). Table 4 summarizes the mean work engagement scores for all combinations of tasks.

**Table 4:** Mean work engagement scores of UMCU faculty for each of three tasks, related to their task combination of employment

Task combination	*N*	All work	Teaching	Research	Patient care
Teaching + research + patient care	151	4.24 (0.84)	3.58 (1.11)	3.65 (1.28)	4.43 (0.82)
Teaching + patient care	25	4.14 (0.85)	3.93 (1.01)	–	4.33 (1.05)
Teaching + research	67	4.35 (0.90)	3.78 (1.18)	4.34 (0.98)	–
Teaching	35	4.42 (0.84)	4.52 (0.82)	–	–
Overall average (SD)		4.26 (0.85)	3.78 (1.13)	3.87 (1.23)	4.42 (0.85)

Values: 1 (low) to 6 (high). Standard deviations between brackets

Teachers combining all three tasks indicated they felt less involved in their educational task than those who only teach or combine teaching with either patient care or research (t = 3.233; df = 268; *p* = 0.001). Those with only a teaching task (mean rank 189.26) felt more involved than teachers who combine tasks (mean rank 127.49). This difference is significant (Z = −4,368; *p* < 0.001).

Table 5 shows the mean teaching engagement scores per division. The high score in the Education Department is in accordance with our finding that teacher engagement is influenced by task combination, as most of the respondents working for this division have no research or patient care tasks. In other divisions, teaching and research are often combined with patient care. The engagement in teaching is not only influenced by task combination but is also division-dependent (Kruskal–Wallis = 30.65; df = 12; *p* < 0.05). An example is the high teaching engagement of physicians in the division of gynaecology, obstetrics and neonatology.

**Table 5:** Mean engagement scores of UMCU faculty for teaching, per division

UMCU divisions (names adapted for clarity)	N = 253	Teaching engagement
Education department	24	4.56 (0.89)
Gynaecology, obstetrics and neonatology	9	4.56 (0.79)
Imaging	9	4.14 (1.01)
Family medicine, public health and epidemiology	27	3.75 (1.23)
Biomedical and genetic sciences	11	3.72 (1.15)
Neuro sciences	31	3.71 (0.85)
Paediatrics	39	3.71 (1.29)
Lab and pharmacy	24	3.70 (1.18)
Lung and cardiac specialities	13	3.58 (1.15)
Internal medicine and dermatology	38	3.49 (1.04)
Surgical specialties	18	3.36 (1.16)
Perioperative and emergency care	10	3.29 (0.54)

Values: 1 (low) to 6 (high). Standard deviations between brackets

### Motivational factors predicting work engagement

The predictive strength of motivational items for work engagement in teaching was investigated using a stepwise multiple regression analysis. The factor feedback on my teaching performance shows a correlation of 0.36 (*p* < 0.01). Adding the factor Possibility of a teaching career resulted in a multiple correlation of 0.45 (*p* < 0.01), adding Teaching large groups (r = 0.51; *p* < 0.01), Teaching with emphasis on the learning process (r = 0.57, *p* < 0.01), Freedom to determine how I teach (r = 0.61, *p* < 0.01), Numerical rating/scores from student evaluations (r = 0.63, *p* < 0.01) and, finally, Wider availability of teacher training provided a multiple correlation of 0.65 (*p* < 0.01). Other items did not further increase this correlation. These seven factors together predicted 42 % of the engagement score for teaching, in which feedback on my teaching performance showed the strongest predictive value (13 % explained variance).

## Discussion

Our study shows that physicians working at a university medical centre score favourably on overall work engagement (with average UWES scores ranging from 3.78 to 4.42). This finding compares with other studies [11–13]. Schaufeli and Bakker [14] report average UWES scores of 3.74 in a large general population, Hof et al. [12], 4.18 among teachers and Prins et al. [13] 4.11 among medical residents. Schaufeli and Bakker [14] designate UWES-9 scores (the version we used) ≤1.77 as very low, 1.78–2.88 as low, 2.89–4.66 as average, 4.67–5.50 as high and ≥5.51 as very high.

Our findings are also consistent with a recent retrospective analysis [15] of data of 9,638 faculty from 23 US schools, showing that about 60–70 % are satisfied with their department, satisfied with their school and indicate that they would choose to work at the same school again if they ‘had to do all over’. However, they are somewhat in contrast with Lowenstein’s report from one school showing that 42 % of faculty consider leaving within 5 years because of dissatisfaction with low recognition of their work, difficult balance of work and family life, little influence on leadership performance, and the absence of faculty development and an academic community [2]. This picture was roughly equal among subgroups of basic science and clinical faculty, among junior and senior faculty and among male and female faculty.

What makes our study interesting is that these overall pictures do not apply when looking to task combination. UMCU teachers find patient care considerably more engaging than teaching and also somewhat more engaging than research. Furthermore, task combinations appear to decrease teaching engagement. Respondents with only a teaching task show a fairly high mean UWES score of 4.52, but when combined with either research or patient care, we find mean scores from 3.78–3.93, and among respondents who combine all three this mean is 3.58.

Based on our data, we can only speculate that dedication to fewer task fields may increase engagement in teaching. Focusing on specific tasks may increase the likelihood of vigour, dedication to the task, and immersion in the work activity. We found some support for this contention in a secondary analysis revealing that teachers who combine all three tasks attach more value to the factor ‘teaching about my speciality’ than teachers with fewer tasks (t = 4.107; df = 243.856; *p* < 0.001). This leads to the hypothesis that teachers with combined tasks may not be able to find sufficient time for teaching and are then mainly satisfied with teaching about content they do not need to prepare. As the combination of teaching, research and patient care is often advocated to work optimally if a physician is in an academic setting, our findings challenge this assumption. The combination of two instead of three tasks in a given period might stimulate motivation in teachers. The price paid is a decrease in all-round clinical faculty within whom research skill feeds into teaching quality and into patient care and vice versa, but we believe that many faculty realize that they cannot excel in all domains, given the high demands for excellence in each of these domains.

Our study further reveals that job resources should include performance feedback, professional growth and autonomy, thus resembling the outcomes of the study of Da Silva Campos Costa [9] on teachers working in higher medical education. It is interesting to see that the top five factors from our survey, marked by the respondents (Teaching about my speciality; Noticeable appreciation for teaching from my direct superior; Teaching small groups (~12 students); Feedback on my teaching performance; Freedom to determine what I teach) perfectly match with the three psychological needs that SDT predicts stimulate intrinsic motivation: feelings of Competence, Autonomy and Relatedness [4]. In addition, motivated students and adequate facilities, planning, organization and support are deemed important. The latter group has been reported before as a significant condition [16]. It is a challenge for a medical school with a centrally directed course with integrated modules to balance between predetermined objectives and regulated course materials and teaching formats on one hand, and freedom for teachers to determine their own content and approach. Our study forces to determine this balance in the best possible way.

Our study has some evident limitations. The fact that the study was carried out in one large university medical centre only limits its generalizability. The local culture at UMCU related to teaching may be different from other medical schools. Our findings do, however, concord with theoretical notions and are supported by earlier findings elsewhere. Second, the response was not optimal. We missed about 50 % of the population sought. If we assume that teachers who are motivated to answer a questionnaire are, on average, also the more motivated teachers, our findings might be somewhat overestimated. But this type of bias may also play a role in other studies that we cited; the comparison of UWES scores therefore might not be greatly affected. We found a rich set of motivating factors as mentioned in response to open questions and we believe we have reached a valid overview of these elements in UMC Utrecht. One final limitation is that our hypothesis that combining tasks lowers motivation and job engagement is not based on experimental data. In other words, teachers who chose to focus on only one or two tasks may have been the more motivated ones in the first place. We cannot exclude this possibility, and would suggest that a study with an experimental design would be needed to confirm our hypothesis.

## Conclusion

This study was conducted to generate input for local curricular and institutional improvement. Next to these local benefits, we believe we have shown more in general how the satisfaction of psychological needs of feelings of competence, autonomy and relatedness, as delineated in SDT [4], may enhance intrinsic motivation in teaching in a University Medical Centre. In curricular and institutional development, motivational factors and conditions might be considered more than we have done in the past [17].

## Essentials

- Teachers in a university medical centre show relatively favourable work engagement.
- If combined with academic tasks, work engagement in patient care is higher than in research and teaching. Combining tasks appears to particularly decrease teaching engagement.
- Factors that teachers indicate to stimulate their motivation include autonomy in determining content of teaching, feedback and being acknowledged for teaching tasks, teaching small groups, motivated students and adequate supporting facilities.
- Of seven motivational factors predicting teaching engagement feedback on teaching performance shows the strongest predictive value.
- Findings are concordant with the job demands–resources model and with self-determination theory.
